# Targeting ER-Mitochondria Signaling as a Therapeutic Target for Frontotemporal Dementia and Related Amyotrophic Lateral Sclerosis

**DOI:** 10.3389/fcell.2022.915931

**Published:** 2022-05-27

**Authors:** Sandra M. Martín-Guerrero, Andrea Markovinovic, Gábor M. Mórotz, Shaakir Salam, Wendy Noble, Christopher C. J. Miller

**Affiliations:** Department of Basic and Clinical Neuroscience, Institute of Psychiatry, Psychology and Neuroscience, King’s College London, London, United Kingdom

**Keywords:** mitochondria, endoplasmic reticulum, amyotrophic lateral sclerosis (ALS), frontotemporal dementia (FTD), therapeutic targets, ER-mitochondria contact

## Abstract

Frontotemporal dementia (FTD) and amyotrophic lateral sclerosis (ALS) are two major neurodegenerative diseases. FTD is the second most common cause of dementia and ALS is the most common form of motor neuron disease. These diseases are now known to be linked. There are no cures or effective treatments for FTD or ALS and so new targets for therapeutic intervention are required but this is hampered by the large number of physiological processes that are damaged in FTD/ALS. Many of these damaged functions are now known to be regulated by signaling between the endoplasmic reticulum (ER) and mitochondria. This signaling is mediated by “tethering” proteins that serve to recruit ER to mitochondria. One tether strongly associated with FTD/ALS involves an interaction between the ER protein VAPB and the mitochondrial protein PTPIP51. Recent studies have shown that ER-mitochondria signaling is damaged in FTD/ALS and that this involves breaking of the VAPB-PTPIP51 tethers. Correcting disrupted tethering may therefore correct many other downstream damaged features of FTD/ALS. Here, we review progress on this topic with particular emphasis on targeting of the VAPB-PTPIP51 tethers as a new drug target.

## Introduction

### Frontotemporal Dementia and Amyotrophic Lateral Sclerosis

FTD is the second most common form of presenile dementia after Alzheimer’s disease and is clinically, genetically and pathologically linked to the most common form of motor neuron disease, ALS. Thus, significant proportions of FTD and ALS patients display features of both diseases (Ringholz et al., 2005; Wheaton et al., 2007). Likewise, both diseases have a genetic overlap and pathogenic variants in the same genes can cause familial dominantly inherited forms of both FTD and ALS (Ling et al., 2013; Robberecht and Philips, 2013; Abramzon et al., 2020). Finally, both diseases can display similar pathological phenotypes and notably, the accumulation of abnormal aggregates of TAR DNA-binding protein 43 (TDP43) in affected neurons (Arai et al., 2006; Neumann et al., 2006).

Like other major neurodegenerative diseases, there are no cures or even effective treatments for either FTD or ALS and so there is much interest in strategies to identify new therapeutic targets. However, a large number of cellular and physiological processes are damaged in FTD/ALS. These include damage to mitochondria, the endoplasmic reticulum (ER), Ca^2+^ signaling, lipid metabolism, autophagy, axonal transport and finally both diseases display inflammatory responses within the nervous system (Paillusson et al., 2016; Lau et al., 2018; Markovinovic et al., 2022). This makes it difficult to select which damaged function to prioritise as a drug target. Recently, alterations to signaling between the ER and mitochondria has been a focus of interest and this is because ER-mitochondria signaling regulates many of the damaged functions seen in FTD/ALS (Paillusson et al., 2016; Lau et al., 2018; Markovinovic et al., 2022). This has led to the notion that targeting the ER-mitochondria axis may be a route to correct many damaged FTD/ALS functions and achieve effective disease modification.

### ER-Mitochondria Signaling Regulates a Broad Number of Physiological Functions

It is now widely accepted that organelles communicate with each other; this permits them to respond dynamically to changes in the cellular environment in an orchestrated manner (Cohen et al., 2018; Gordaliza-Alaguero et al., 2019). Communications between the ER and mitochondria represent a particularly important component of organelle signaling since this regulates several key cellular processes. These include bioenergetics, Ca^2+^ homeostasis, lipid metabolism, mitochondrial biogenesis and trafficking, apoptosis, ER stress responses, autophagy and inflammation (Rowland and Voeltz, 2012; Krols et al., 2016; Paillusson et al., 2016; Csordas et al., 2018; Rieusset, 2018; Perrone et al., 2020; Markovinovic et al., 2022). Additionally, in neurons ER-mitochondria signaling regulates synaptic activity and damage to synaptic function is a defining feature in neurodegenerative diseases including FTD/ALS (Herms and Dorostkar, 2016; Hirabayashi et al., 2017; Spires-Jones et al., 2017; Gomez-Suaga et al., 2019).

**FIGURE 1:**
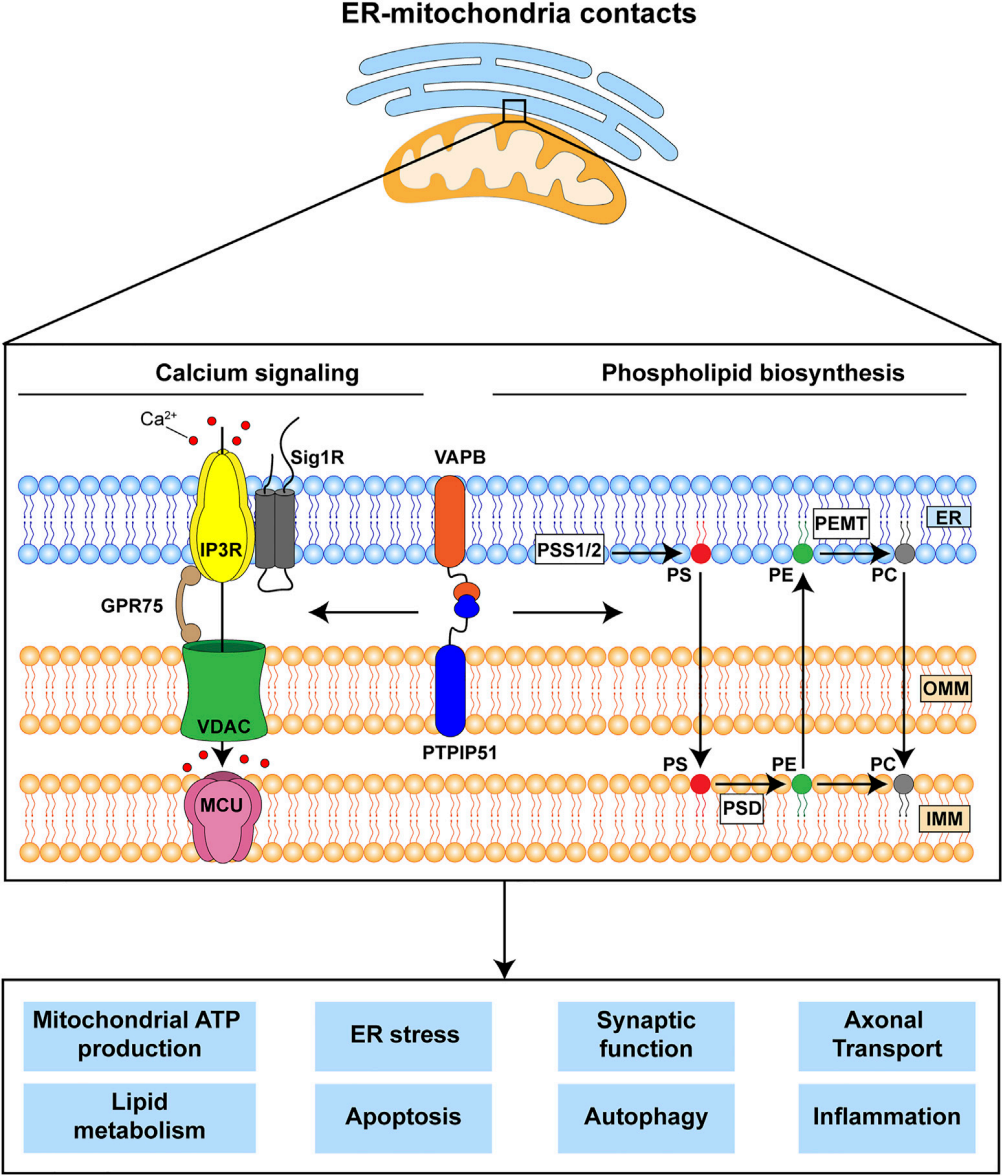
The VAPB-PTPIP51 tethers regulate delivery of Ca^2+^ from ER stores to mitochondria and phospholipid synthesis. These primary functions are believed to impact upon a number of other downstream physiological processes many of which are damaged in FTD/ALS. The VAPB-PTPIP51 interaction facilitates Ca^2+^ transfer from ER to mitochondria *via* IP3R-GRP75-VDAC1. Phospholipid synthesis involves initial production of phosphatidylserine (PS) in MAM by PS synthase 1 and 2 (PSS1/2); this is transferred to mitochondria where PS decarboxylase (PSD) converts it to phosphatidylethanolamine (PE). PE can be transferred back to the ER, where phosphatidylethanolamine N-methyltransferase (PEMT) converts it to phosphatidylcholine (PC). Finally, PC can be also transferred back to mitochondria. ER, endoplasmic reticulum; IMM, inner mitochondrial membrane; IP3R, inositol 1,4,5-trisphosphate receptor; GPR75, glucose-regulated protein 75; MCU, mitochondrial Ca^2+^ uniporter; OMM, outer mitochondrial membrane; Sig1R, Sigma-1 receptor; VDAC1, voltage-dependent anion-selective channel.

The mechanisms by which ER-mitochondria communications impact on all these different cellular processes are not properly understood but the two primary functions of ER-mitochondria signaling are delivery of Ca^2+^ from ER stores to mitochondria and the synthesis of phospholipids (Rowland and Voeltz, 2012; Vance, 2015; Paillusson et al., 2016; Csordas et al., 2018; Markovinovic et al., 2022). It is likely that these primary functions impact on the other downstream roles of ER-mitochondria signaling (Figure 1). Mitochondria require Ca^2+^ to generate ATP and this is because dehydrogenases in the tricarboxylic acid cycle are Ca^2+^ dependent. In addition, mitochondrial Ca^2+^ is involved in the activation of Ca^2+^-regulated mitochondrial carriers (CaMCs) located in the inner mitochondrial membrane (IMM) (Del Arco et al., 2016). The major route for delivery of this Ca^2+^ involves its release from ER stores *via* inositol 1,4,5-trisphosphate (IP3) receptors and uptake into mitochondria by the outer mitochondrial membrane located voltage-dependent anion-selective channel-1 (VDAC1) and the inner membrane located mitochondrial calcium uniporter (MCU) (Rowland and Voeltz, 2012; Paillusson et al., 2016; Csordas et al., 2018). As such, ER-mitochondria signaling regulates bioenergetics and indeed, changes in metabolic demand have been shown to stimulate ER-mitochondria signaling (Gomez-Suaga et al., 2019) (Figure 1). Aside from its release from IP3 receptors, Ca^2+^ can also be released from Ryanodine receptors in ER for uptake by mitochondria (Csordas et al., 2018). There are also several other subunits to the MCU channel; together these other proteins can all influence ER-mitochondria Ca^2+^ exchange (Feno et al., 2021).

The second primary function is to synthesise phospholipids. Although most phospholipids are produced in the ER, some are synthesised by enzymes that are located in both ER and mitochondria; for these, precursor exchange between the two organelles is required. Indeed, two of the most abundant phospholipids in mammalian cells, phosphatidylcholine and phosphatidylethanolamine are produced at the ER-mitochondria axis (Rowland and Voeltz, 2012; Vance, 2015; Paillusson et al., 2016; Csordas et al., 2018) (Figure 1).

### ER-Mitochondria Tethering Proteins

ER-mitochondria signaling involves close physical contacts between the two organelles such that up to approximately 20% of the mitochondrial surface is closely apposed (distances of about 10–30 nm) to ER membranes (Csordas et al., 2006; Paillusson et al., 2016; Csordas et al., 2018; Markovinovic et al., 2022). These regions of ER are termed mitochondria-associated ER membranes (MAM). The mechanisms by which these contacts form are not fully understood but it is generally agreed that it involves “tethering” proteins that serve to recruit regions of ER to the mitochondrial surface. A number of different tethers have now been described and it is possible that different tethers serve to recruit different domains of ER to mitochondria e.g., rough and smooth, and sheets and tubules of ER; also there are proteins that act to regulate the interactions of tethers and their functions. Such tethers and regulators have recently been reviewed (Csordas et al., 2018; Markovinovic et al., 2022). The tethering proteins most strongly linked to FTD/ALS involve an interaction between the integral ER protein vesicle-associated membrane protein-associated protein B (VAPB) and the outer mitochondrial membrane protein, protein tyrosine phosphatase interacting protein-51 (PTPIP51) (De Vos et al., 2012; Stoica et al., 2014). The VAPB-PTPIP51 tethers regulate IP3 receptor mediated delivery of Ca^2+^ to mitochondria, phospholipid synthesis and synaptic activity (De Vos et al., 2012; Gomez-Suaga et al., 2019; Yeo et al., 2021).

### A Number of Genetic Insults That Cause Familial Forms of FTD/ALS Disrupt ER-Mitochondria Signaling and the VAPB-PTPIP51 Tethers

**FIGURE 2:**
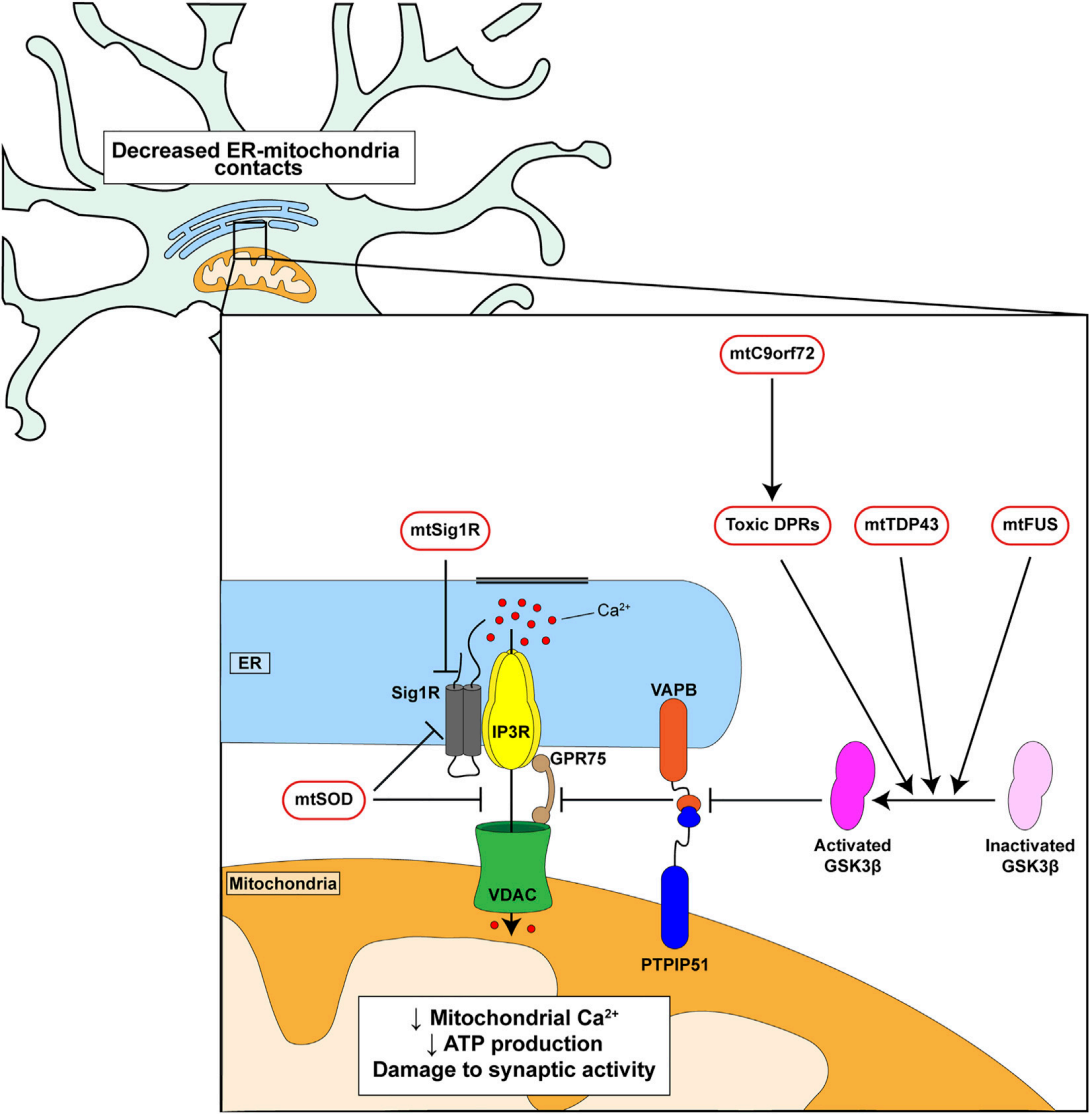
Genetic insults linked to familial FTD/ALS disrupt ER-mitochondria signaling and the VAPB-PTPIP51 interaction. *C9orf72*-derived toxic DPRs, mutant TDP43 and mutant FUS all activate GSK3*β* which in turn disrupt binding of VAPB to PTPIP51. Mutant Sigma1 receptor perturbs IP3 receptor mediated delivery of Ca^2+^ from ER to mitochondria. Mutant SOD1 may act directly on the Sigma-1 receptor and/or target the IP3 receptor-VDAC1 interaction.

A number of genes have now been identified as causal for familial inherited forms of FTD/ALS (Abramzon et al., 2020). Several of these have been shown to disrupt ER-mitochondria contacts and/or mitochondrial Ca^2+^ delivery. These include mutant *SIGMAR1* encoding the Sigma-1 receptor, mutant *SOD1* encoding Cu/Zn superoxide dismutase-1 (SOD1), mutant *TARDBP* encoding TDP43, mutant *FUS* encoding fused in sarcoma and mutant *C9orf72* (Figure 2) (Stoica et al., 2014; Bernard-Marissal et al., 2015; Dafinca et al., 2016; Gregianin et al., 2016; Stoica et al., 2016; Watanabe et al., 2016; Dafinca et al., 2020; Gomez-Suaga et al., 2022). The Sigma-1 receptor is an ER protein that functions as a chaperone for IP3 receptors to facilitate delivery of Ca^2+^ to mitochondria; the disease-causing alterations are loss of function mutations (Bernard-Marissal et al., 2015; Gregianin et al., 2016; Watanabe et al., 2016). Mutant SOD1 damages ER-mitochondria signaling *via* disruption of Sigma-1 receptor function (Watanabe et al., 2016). TDP43 accumulations form the hallmark pathology of FTD/ALS but FUS is now also known to be a widespread pathology of FTD/ALS (Spires-Jones et al., 2017; Tyzack et al., 2019). Mutations in the *C9orf72* gene cause most familial FTD/ALS cases (Dejesus-Hernandez et al., 2011; Renton et al., 2011). The mutations involve expansion of an intronic hexanucleotide repeat which is translated into dipeptide repeat (DPR) proteins, some of which have been shown to be neurotoxic (Kwon et al., 2014; Mizielinska et al., 2014; Wen et al., 2014).

Mutant *C9orf72*, TDP43 and FUS all disrupt ER-mitochondria contacts and Ca^2+^ exchange *via* an effect on the VAPB-PTPIP51 tethers. For *C9orf72* this involves the toxic DPRs (Figure 2) (Stoica et al., 2014; Stoica et al., 2016; Gomez-Suaga et al., 2022). Moreover, this breaking of the VAPB-PTPIP51 tethers is an early feature that appears before disease onset in mutant *C9orf72* transgenic mice; early pathogenic changes are believed to be the most important so this finding supports the notion that disruption of the VAPB-PTPIP51 tethers contributes in a major way to disease (Gomez-Suaga et al., 2022). In addition, damage to the VAPB-PTPIP51 tethers by mutant TDP43, FUS and *C9orf72*-derived DPRs involves activation of glycogen synthase kinase-3*β* (GSK3*β*) (Stoica et al., 2014; Stoica et al., 2016; Gomez-Suaga et al., 2022) (Figure 2). GSK3*β* is a modulator of the VAPB-PTPIP51 interaction; activation disrupts whereas inhibition stimulates binding (Stoica et al., 2014; Stoica et al., 2016). GSK3*β* is strongly implicated in dementia and ALS and so GSK3*β* inhibitors may prove to be therapeutic for FTD/ALS (Llorens-Martin et al., 2014; Lauretti et al., 2020). Thus, disruption of ER-mitochondria contacts and signaling is a feature of several familial FTD/ALS linked genes and where studied, this involves breaking of the VAPB-PTPIP51 tethers.

### ER-Mitochondria Signaling as a Drug Target for FTD/ALS

The findings that several FTD/ALS linked genes disrupt ER-mitochondria signaling and that this signaling regulates many damaged functions, suggests that correcting this disruption may remedy other downstream disease features (Paillusson et al., 2016) (Figure 1). Enhancing ER-mitochondria contacts and signaling may therefore be broadly therapeutic. GSK3*β* inhibitors provide an obvious solution but whilst a number of these have shown beneficial effects in disease models, none have so far made it to the clinic as treatments for neurodegenerative disorders. This may be because GSK3*β* has functions outside of the nervous system and inhibition of these other functions may be detrimental. Another route involves use of Sigma-1 receptor agonists since loss of *SIGMA1R* causes familial FTD/ALS (Bernard-Marissal et al., 2015; Gregianin et al., 2016; Watanabe et al., 2016). Several Sigma1 receptor agonists have proved to be beneficial in cell and animal models of neurodegenerative diseases including FTD/ALS and one, Anavex2-73 is being tested in clinical trials (Watanabe et al., 2016; Ryskamp et al., 2019). A further route involves identifying novel agents that stimulate ER-mitochondria contacts and screens for the identification of such molecules are discussed below. However, it must be stressed that a strong reinforcement of ER-mitochondria tethering is likely to be detrimental to neurons as it could induce Ca^2+^ overload in mitochondria which can be a signal for apoptosis (Paillusson et al., 2016; Csordas et al., 2018; Markovinovic et al., 2022).

### Assays for Monitoring the Strength of ER-Mitochondria Tethering, Contacts and Signaling

A number of cellular assays for monitoring the strength of ER-mitochondria contacts and signaling have been devised and reported. The first involves use of split or dimer-dependent fluorescent proteins such as enhanced green fluorescent protein (EGFP). Here, split or dimer dependent EGFP moieties are directed to ER and mitochondria respectively *via* ER and mitochondria targeting sequences. Close associations between the EGFP moieties at MAM analogous to those seen in fluorescence resonance energy transfer (FRET) assays generate signals which can be quantified after application of potential therapeutics to the media in drug screens (Alford et al., 2012; Cieri et al., 2018; Kakimoto et al., 2018; Yang et al., 2018; Calì and Brini, 2021). Such assays have already facilitated the identification of the flavonoid luteolin as a stimulator of ER-mitochondria contacts; luteolin was identified *via* its ability to stimulate mitochondrial ATP production but a secondary split-EGFP assay was used to show it influences ER-mitochondria contacts (Naia et al., 2021).

An extension of such cellular assays involves monitoring how potential therapeutics might influence the interaction of known tethering proteins involved in FTD/ALS such as VAPB and PTPIP51. Here, split or dimer dependent EGFP moieties are fused to VAPB and PTPIP51 and signals again quantified after application of drugs to the media. Similar approaches could involve bioluminescence resonance energy transfer (BRET) assays such as Nanoluc Binary Technology (NanoBiT) luciferase complementation assays (Dale et al., 2019). Nanoluc is derived from *Oplophorus gracilirostris* (deep sea shrimp) luciferase and is genetically engineered for minimal size and optimal performance in luciferase assays. Complementation assays to monitor the strength of protein-protein interactions involve fusion of fragments of Nanoluc (LargeBiT and SmallBiT) to the proteins of interest (VAPB and PTPIP51). Readouts for NanoBiT assays are performed without cell lysis so the signals obtained represent the strength of protein-protein interaction in living cells.

Finally, proximity ligation assays (PLAs) can be used quantify the strength of ER-mitochondria contacts and the VAPB-PTPIP51 interaction in drug screens. The distances detected by proximity ligation assays are similar to those detected by FRET (i.e., approximately 30 nm) (Soderberg et al., 2006). Such proximity ligation assays have already been used to quantify ER-mitochondria contacts and binding of VAPB to PTPIP51 (De Vos et al., 2012; Hedskog et al., 2013; Stoica et al., 2014; Bernard-Marissal et al., 2015; Stoica et al., 2016; Gomez-Suaga et al., 2019; Gomez-Suaga et al., 2022).

One disadvantage of the above cellular assays is that the primary target of any novel drug is not clear. For example, it could be the VAPB-PTPIP51 interaction itself or some upstream regulator such as GSK3*β*. Deconvolution of the mechanism of action of any identified drug is thus required and this can be time consuming.

As an alternative to the above cellular assays, *in vitro* binding assays with purified recombinant tethering proteins such as VAPB and PTPIP51 can be employed to screen for small molecules that enhance the interaction. Clearly, such assays only identify molecules that act directly on the tethers so further deconvolution work is less labour intensive. One route would be to use FRET based methods to monitor the strength of the VAPB-PTPIP51 interaction *in vitro* after application of drug. As an extension, fragment-based drug discovery methods could also be applied. Traditional small molecule drug screens involve use of millions of compounds but fragment-based screens utilise much smaller libraries containing very low molecular mass molecules termed “fragments”. These have low complexity which can enable them to bind to key areas of the protein(s) of interest. Once lead “fragments” have been identified, they can then undergo medicinal chemistry to increase affinity and biological potency (Erlanson et al., 2016). Modulating protein-protein interactions is considered a relatively difficult drug target but the use of fragment based methods is enabling rapid progress in this area (Modell et al., 2016; Valenti et al., 2019).

## Discussion

New drug targets for FTD/ALS are required and ER-mitochondria signaling represents a particularly attractive one. This is because: 1) ER-mitochondria signaling is damaged in FTD/ALS and where studied is an early disease feature; early pathogenic changes are believed to be the most important. 2) ER-mitochondria signaling regulates many of the other damaged features of FTD/ALS so correcting this damage may be broadly beneficial. 3) VAPB and PTPIP51 have been identified as ER-mitochondria tethers that are disrupted in FTD/ALS (Stoica et al., 2014; Bernard-Marissal et al., 2015; Dafinca et al., 2016; Gregianin et al., 2016; Stoica et al., 2016; Watanabe et al., 2016; Dafinca et al., 2020; Gomez-Suaga et al., 2022). The VAPB-PTPIP51 interaction thus represents a defined molecular target for drug intervention.

Interestingly, damage to ER-mitochondria tethering and signaling has also been described for other neurodegenerative diseases including Alzheimer’s disease and Parkinson’s disease (Paillusson et al., 2016; Markovinovic et al., 2022). However, for these diseases there is evidence that damage may involve increased or reduced ER-mitochondria contacts and signaling (Paillusson et al., 2016; Markovinovic et al., 2022). Thus, it is possible that inhibitors of ER-mitochondria tethering may also have therapeutic potential. Whatever the precise mechanism, the screens described above may be beneficial in identifying such molecules.
